# Improving the healthcare user experience: an optimization model grounded in patient-centredness

**DOI:** 10.1186/s12913-024-11960-7

**Published:** 2025-01-23

**Authors:** Elisa Peruzzo, Chiara Seghieri, Milena Vainieri, Sabina De Rosis

**Affiliations:** Management and Healthcare Laboratory, Institute of Management, Sant’Anna School of Advanced Studies, Pisa, Italy

**Keywords:** Patient satisfaction, Patient experience, Hospitalization, Optimization Model, Healthcare service, User-centricity, Quality improvement

## Abstract

**Background:**

Patient satisfaction and experience are key outcomes of healthcare and can be computed as powerful measures of service quality. Understand what affects them is essential for service quality improvement. Investigating whether the care setting (i.e., medical or surgical) can impact the patients’ perception of the quality can be also important for the actionability of this data. The aim is to explore which experiential factors should be prioritized to improve patient satisfaction with hospitalization service, using experience items as intermediate results and considering different settings.

**Methods:**

Patient-reported experience measures are used in an Italian region. This study uses the optimization approach to identify factors of healthcare user experience affecting and enhancing satisfaction.

**Results:**

The results confirm that, among the significant determinants of satisfaction, some specific experiential aspects emerged as the potential primary focus to be prioritized in improvement actions. These aspects vary according to the specific departmental area.

**Conclusions:**

The study presents an optimization model directly informed by healthcare service users, utilizing their insights to drive healthcare delivery improvements. It emphasizes the necessity of not only collect patient perspectives but also applying different methodologies to understand what matters to patients and what interventions could be prioritized, and to strategically use diverse insights to enhance the delivery of healthcare services and patient experience and satisfaction.

## Background

Despite the ongoing debate over whether to measure patient satisfaction or experience, both have become important measures of hospital performance [1]. Historically, patient satisfaction was often prioritized, but in recent years there is a growing recognition of the importance of focusing on patient experience. The distinction between satisfaction and experience is based on the understanding that while satisfaction is a subjective outcome shaped by expectations, patient experience provides more objective, actionable insights into specific aspects of care and into patient values, needs, and preferences [1, 2]. Thus, experience and satisfaction measures provide distinct insights to healthcare managers, professionals and researchers and can serve different purposes. Both are increasingly presented as outcomes, as they reflect the effectiveness of healthcare services and their alignment with patient-centred care [3]. As such, these measures were progressively integrated into multidimensional Performance Evaluation Systems, as process, output and outcome measures accordingly to what they are measuring [4]. Patient-indicators have been employed for benchmarking and accreditation purposes since the initial stages of the evolution of the healthcare performance management models [5–7]. Particularly, Patient-Reported Experience Measures (PREMs) have been widely studied in relation to their potential in moving the focus of healthcare services’ provision towards a more user-centered approach [8–12]. In this sense, PREMs allow identifying and describing different aspects of experience in healthcare services. Surveys and real-time experience monitoring can be instrumental in evaluating the quality of experience with services at different touchpoints throughout their journey [13]. These tools help in the continuous assessment, maintenance, and enhancement of the quality of the user experience, offering a practical and actionable perspective [14].

The data derived from patients’ reported experiences can be employed for different purposes, such as improving hospital comfort (e.g., food service, cleanliness, and noise), enhancing accountability and transparency, fostering effective communication and relationships between patients/caregivers and hospital staff, and informing initiatives targeted at healthcare professionals (e.g., training, improvement of staff motivation and internal communication) [15–20].

Previous studies have indicated that several factors influence data reported by patients on their experiences and satisfaction with healthcare services. These factors include whether the questionnaire was completed individually or with the assistance of someone else, patient characteristics (such as age, sex, and level of education), service-related attributes (like the length of hospital stay or the institutional characteristics of the provider), the method of feedback provision (online or paper-based survey, personal or phone interview), and the external context during participation (e.g., economic crises, pandemics) [21–23]. Some authors have emphasized the importance of distinct elements affecting the inpatient satisfaction, including interpersonal and relational aspects of care, effective communication of information, the organizational care model encompassing nursing, amenities, and privacy [24, 25]. Additionally, other researchers have demonstrated that factors leading to the most substantial increase in satisfaction in the surgical setting encompass respectful treatment by nurses and doctors, clear nursing and medical explanations, proficient pain management, cleanliness of spaces, and prompt assistance [26].

The departmental area (i.e., surgical and medical) is associated with the patients’ perception of the quality of care [27]. Murante et al. (2014) underlined differences between patients admitted in medical and surgical wards: the first are generally older and more likely to suffer from chronic conditions, compared to those in surgical departments [22]. These groups also vary in their rates of patient leaving hospital against medical advice (PLHAMA), which is associated with patient satisfaction and with other performance metrics, such as outcomes (30-day mortality) [22, 28]. This latter association is significant for surgical admissions, whereas it is not for medical ones. At the same time, within medical pathways, hospitalization may represent a distinct and specific stage within a longer, complex, and multi-provider pathway, thus warranting separate investigation.

Users’ satisfaction is therefore a powerful measure of service quality [29], however using satisfaction as a quality metric is paradoxical if the factors influencing patient satisfaction are not fully understood and taken into consideration into the quality improvement actions [26]. Acquiring patient assessments is imperative for enhancing care quality initiatives and comprehending the aspects of care that hold significance for patients [15]. Nonetheless, to the best of our knowledge, there are no studies in the literature investigating the impact of different aspects of healthcare service experiences on overall patient satisfaction with hospitalization, using an optimization model. Previous studies using patient-reported measures to identify the best possible combination of predictors of overall patient satisfaction focused on other healthcare settings, i.e., emergency department [30–32], nursing homes [33], cancer care [34]. Further research is necessary to identify which factors should be prioritized to enhance the experience with hospital stay from the patient’s perspective and considering differences related to the different setting of hospitalization experience.

This study treats experience items as intermediate results to streamline the optimization model and examine the primary factors influencing overall satisfaction in surgical and medical departments. This does not imply that patient experience is not meant as outcome in this study, since this model just helps to identify primary experiential aspects for prioritize quality improvement actions. Patient-reported data is usually skewed towards positive evaluations. This can lead to lower trust in data and a perception among healthcare managers and professionals that improvements can only be marginal [20, 35]. This perception may undervalue the utility of such data, highlighting the need for trust in its accuracy and actionability, as a crucial factor for effective use in decision-making. Since patient satisfaction is typically higher than patient experience [9], it is useful to pinpoint specific experiential areas for targeted enhancement. Therefore, first we have investigated the experiential factors affecting satisfaction, then we included them into an optimization model for identify the experiential areas of intervention to be prioritized. As reported by Seghieri and colleagues (2009), this methodology seems to possess generalizability and applicability across various settings where patient satisfaction data are gathered, albeit with a caveat that it is sensitive to the specific context [30].

Based on these premises, our aim is to investigate which possible aspects of hospitalization experience affect the most patient satisfaction with hospitalization in different departmental areas, with a technique able to capture what can be prioritized. In a resource-constrained environment, prioritizing interventions becomes essential. Incorporating patient feedback into quality improvement efforts can be resource-intensive and is often seen as a barrier to using patient-reported data. This study’s optimization model identifies key aspects that influence patient satisfaction, demonstrating that such data can provide actionable insights for managing healthcare services more effectively. By understanding priority areas, healthcare professionals can better allocate their time and develop targeted quality improvement initiatives.

Moreover, this study emphasizes the need for context-specific actions, advocating for data dissemination and analysis at multiple organizational levels. In fact, our setting are seven public hospitals of an Italian region (i.e., Tuscany Region). We focused on the experiences of patients discharged from surgical and medical wards. This distinction was made for exploring whether the care setting has an impact on the results of the optimization model since we expected different aspects of care to be prioritized in different settings, in efforts to enhance patient satisfaction.

## Methods

### Data collection

In this research, we used data from the PREMs (Patient-Reported Experience Measures) Observatory, an ongoing and continuous system for collecting patient self-reported feedback on their hospitalization experiences and reporting them to practitioners [36, 37]. Within Tuscany Region, 3 Local Health Authorities (LHAs), 4 Teaching Hospitals (THs) and a children’s hospital (CH) joined the PREMs Observatory since 2019, for a total of 8 hospitals.

For this study, we focused on the experience reported by patient discharged in 2022. This approach was chosen due to the challenges in comparing years marked by different events, such as the Covid-19 pandemic. Additionally, we concentrated on the experiences and satisfaction of adult patients, excluding the results from the children’s hospital, due to the different target of patients.

In 2022, more than 27,700 patients provided their feedback via the online questionnaire of the PREMs Observatory. Table 1 reports the distribution of responding patients per departmental area, according to the categorization illustrated into the Decree of the Minister of Health of 5th December 2006, Annex 1: “*Codes of clinical and hospital specialities*”. In this study, we focused exclusively on surgical and medical department for the highest number of responses in the observation period, for a total of 24,162 observations.

**Table 1:** Number of responding patient per departmental areas of discharge (2022)

Departmental areas	Responding patient (n°)
Surgical	14,975
Medical	9,187
Traumatological orthopaedic	2,867
Emergency	109
Other disciplines	1,596
TOTAL	28,734

The PREMs questionnaire is composed by items of experience and satisfaction with hospitalization service, in addition to questions on socio-demographic characteristics (e.g., sex, age, level of education). The complete questionnaire is available in De Rosis et al. (2020) [36].

The experience items were assessed with a 5-point Likert scale (i.e., 1 = never, 2 = rarely, 3 = sometimes, 4 = often and 5 = always). The items used in the study included the following: kindness during hospital reception; management of fear and anxiety by doctors and by nurses; pain management; talk in front of the patient as if s/he was not present by doctors, nurses and other hospital staff; respect and dignity demonstrated by doctors, nurses and other hospital staff; patient involvement in decision-making; clarity of answer by doctors and nurses; involvement of the family members or caregivers; perceived teamwork; silence in the department; cleanliness of the department; clear answers at discharge about selfcare (e.g., sports, food, smoking) and therapy (i.e., drugs).

The item of overall satisfaction that we used in this study was: ‘‘*Overall, how would you rate the care you received in the department?*’’. This item is also evaluated using a 5-point Likert scale (i.e., 1 = very poor, 2 = poor, 3 = sufficient, 4 = good and 5 = excellent).

Patient-reported data shows a tendency towards positive evaluations. It often tends to show higher value for general satisfaction compared to specific patient experience measures, as highlighted by Coulter et al. [9]. Therefore, we treated experience measures as intermediate outcomes to target for enhancing overall satisfaction. Identifying specific experiential areas where professionals have the greatest potential for quality improvement is crucial for effective, targeted enhancement of healthcare services’ quality.

### Data analysis

We performed descriptive statistics of the socio-demographic variables.

Afterwards, we apply an optimization method to PREMs data to identify the best (most efficient) combination of aspects to drive a preset level of improvement in overall satisfaction. An optimization model is a mathematical framework used to find the best possible solution from a set of feasible alternatives, considering specified conditions [34]. An optimization model is applied to make informed decisions by maximizing or minimizing a specific objective while adhering to certain constraints. Other studies used the optimization model since it allowed researchers to uncover patterns, trends, and relationships among variables that might not be immediately apparent through traditional analytical methods, so providing a structured and objective approach to making healthcare data-driven decisions [22, 33, 38].

Following the methodology employed by Brown et al. (2005) and Sandoval et al. (2006), we performed multiple ordinal logistic regression models to estimate relationships between independent variables (i.e., aspects of care experience) and dependent variables (i.e., overall satisfaction). Two regressions were performed: one for each departmental area. The regression coefficients were then integrated into a optimization model to identify the optimal solutions capable of increasing the overall satisfaction by a maximum of 15% [31, 34]. The optimization model allows to select the most efficient combination of independent variables based on the current performance of the predictors and the strength of the predictors [31].

Statistical analyses were performed using STATA software and Excel Solver.

## Results

In the Table 2, we reported the descriptive analysis of socio-demographic characteristics of patients participating to the PREMs Observatory in 2022. We differentiated between the surgical and medical areas.

**Table 2:** Socio-demographic characteristics of the sample of respondents per departmental areas of discharge

		Surgical Area		Medical area		*P*value
		N**°**	%	N°	%	
Gender	Female	8,127	64.2	2,975	44.3	0.000
	Male	4,542	35.8	3,736	55.7	
Age	< 18	34	0.2	512	5.6	0.000
	18–44	4,073	27.2	527	5.7	
	45–64	7,336	49.0	3,168	34.5	
	> = 65	3,527	23.6	4,980	54.2	
Education Level	Low	4,372	34.5	3,897	58.1	0.000
	Medium	5,009	39.6	1,933	28.8	
	High	3,285	25.9	879	13.1	
Perceived state of health	Bad	241	1.90	769	11.47	0.000
	Sufficient	2,964	23.39	2,510	37.45	
	Good	5,101	40.26	2,195	32.75	
	Very good	3,422	27.01	959	14.31	
	Excellent	942	7.43	269	4.01	
Chronic condition	No	7,866	62.09	1,943	28.97	0.000
	Yes	4,143	32.7	4,251	63.37	
	I don’t know	660	5.21	514	7.66	
Type of hospitalization	Urgent (emergency room)	672	4.78	2,772	34.59	0.000
	Planned	13,374	95.22	5,241	65.41	
Average Length of Stay (days)		3.67		8.15		0.000

Note: The number of observations varies among items due to the voluntary nature of patient participation, allowing them the freedom to discontinue the online questionnaire at any point of the survey

There are more female respondents among patients discharged from the surgical area compared to the medical area (64.2% and 44.3% respectively). The population of respondents from the surgical department is younger (*Mean: 53, SE: 0.1249*) and with a medium level of education. Conversely, in the medical department, patients are older (*Mean: 64.5, SE: 0.2198*) and had a lower level of education. About the health condition of participants, the self-reported degree of chronicity reveals that respondents discharged from the medical area are more likely to be chronic patients compared to the surgical area. In both setting, the majority of responding patients have a planned admission, but with a different distribution: while in surgical area nearly all patients had a planned hospitalization, the 35% of respondents from medical department said they had access to the emergency department before being hospitalized. Finally, there are also differences in the length of stay. In the medical department, the duration of admission is grater compared to the surgical area.

Patient satisfaction with hospitalization was generally positive. On a scale ranging from 1 (lower) to 5 (higher), the mean of overall satisfaction in surgical area was 4.6 (*SE: 0.0059*), while in medical area was 4.5 (*SE: 0.0097*). The difference between examined areas is statistically significant (*P* = *0.000).*

The Table 3 displays the results of the Ordinal Logistic Regression models on the patients’ satisfaction, categorized by surgical and medical area. In the surgical area, all the experience-related items showed a positive and significant relation with patients’ satisfaction, with a few exceptions. These exceptions included the management of fear and anxiety by doctors; talking in front of the patient as if s/he was not present by doctors, nurses and other staff; respect and dignity demonstrated by other staff; and clarity of answers provided by doctors. Conversely, in the medical area, we found a lower number of experiential aspects that exhibited a statistically significant positive relation with the overall satisfaction. Specifically, these aspects were kind reception; pain management; respect and dignity demonstrated by doctors; patient involvement; perception of teamwork; cleanliness; clarity of information provided during the discharge about selfcare and therapy.

**Table 3:** Ordinal logistic regression results for patients’ satisfaction per departmental areas of discharge (*P* = 0.05)

	Surgical Area			Medical Area		
	b	SE	p	b	SE	p
Kind reception	.2859	.059	0.000	.4153	.1047	0.000
Fear and anxiety management—doctors	.0753	.0553	0.174	.0412	.0986	0.676
Fear and anxiety management—nurses	.3086	.06	0.000	.0938	.0948	0.322
Pain management	.5542	.065	0.000	.4682	.1094	0.000
Talk in front of the patient as if he were not present—doctors	.0202	.0596	0.735	.0632	.0985	0.521
Talk in front of the patient as if he were not present—nurses	.0707	.0698	0.311	-.1038	.1249	0.406
Talk in front of the patient as if he were not present – other staff	.0474	.0622	0.446	.1087	.1028	0.290
Respect and dignity—doctors	.2615	.0913	0.004	.3905	.1497	0.009
Respect and dignity—nurses	.245	.0975	0.012	.0351	.1628	0.829
Respect and dignity – other staff	.1049	.0619	0.090	.1501	.099	0.131
Involvement	.1917	.0511	0.000	.1516	.0766	0.048
Clarity of answers—doctors	-.083	.0807	0.304	-.1613	.1355	0.234
Clarity of answers—nurses	.1717	.0787	0.029	.1613	.1265	0.202
Information to caregivers	.1662	.0382	0.000	.1463	.0766	0.056
Teamwork	1.698	.0742	0.000	1.931	.1247	0.000
Silence	.1973	.0453	0.000	.0856	.0775	0.270
Cleaning	-.5276	.0527	0.000	-.5097	.0911	0.000
Clarity of answers in discharge—selfcare	.4815	.091	0.000	.4889	.159	0.002
Clarity of answers in discharge—therapy	.2377	.1041	0.022	.44361	.1721	0.010
/cut1	7.842	.5219		5.848	.817	
/cut2	10.83	.4998		10.57	.801	
/cut3	14.71	.5563		14	.889	
/cut4	19.44	.6269		18.83	1.008	
Observations (n°)	5,434			1,867		
Log-likelihood	−2226.151			−806.513		

We presented the results of the optimization models in the following tables. Specifically, we provided information on the total number of variables (i.e., care aspects) required to enhance satisfaction from 1 to 5% (Table 4) and the associated care aspects (Table 5).

**Table 4:** Total number of experience-related items required to improve the patient satisfaction from 1 to 5%

	Surgical area						Medical area					
Satisfaction improvement (%)	1	2	3	4	5	6	1	2	3	4	5	6
Required variables (n°)	3	4	5	6	9	Unfeasible	2	3	4	5	10	Unfeasible

**Table 5:** The required experience-related items for each unit of satisfaction improvement (1 to 5)

	Surgical area					Medical area				
Satisfaction improvement (%)	1	2	3	4	5	1	2	3	4	5
Kind reception	15	15	15	15	15	12.5	15	15	15	15
Fear and anxiety management – doctors					15					15
Fear and anxiety management – nurses	9.4	15	15	15	15					15
Pain management	15	15	15	15	15	15	15	15	15	15
Talking in front of the patient as if s/he was not present – doctors										15
Talking in front of the patient as if s/he was not present – nurses					8.9					
Talking in front of the patient as if s/he was not present – other staff									2.8	15
Respect and dignity – doctors		8.4	15	15	15		6.8	15	15	15
Respect and dignity – nurses			8.2	15	15					4.6
Respect and dignity – other staff					15			4	15	15
Involvement				11.6	15					15
Clarity of answers – doctors										
Clarity of answers – nurses										
Information to caregivers										
Teamwork										
Silence										
Cleaning										
Clarity of answers in discharge – selfcare										
Clarity of answers in discharge – therapy										

These results are presented separately for the surgical and medical departments. An increase of more than 5% was found to be unfeasible in both the departmental areas due to the high value of overall satisfaction reported by the patients.

Table 5 indicates which experience items need to be prioritized to improve the patient satisfaction (i.e., rows), the necessary percentage of improvement for each item (as indicated by the number in the cell), and the outcome derived by the item improvement reported as increase of patient satisfaction ranging from 1 to 5% (as shown in the columns).

To achieve a 1% increase in overall satisfaction, professionals in the surgical department should consider addressing three specific care aspects: kind reception, management of fear and anxiety by nurses, and pain management. In the medical department, the same improvement can be achieved by focusing on two key aspects of hospital stay: kind reception and pain management.

For a 2% increase in overall satisfaction, the hospital staff in the surgical area should concentrate on four factors. These include the three aspects mentioned earlier, as well as demonstrating respect and dignity by doctors. Similarly, in the medical area, a 2% increase in patient satisfaction necessitates emphasizing three aspects: the two aspects previously mentioned, along with respect and dignity demonstrated by doctors, as in the surgical area.

Aiming for a 3% increase in satisfaction requires focusing on respect and dignity demonstrated by nurses in the surgical setting, and by other staff in the medical area, in addition to the above-mentioned four and three aspects respectively to be prioritized in the experience with hospitalization.

To achieve a 4% increase in patient satisfaction, improvement strategies in surgical wards should also consider patient involvement, while in medical wards addressing the behaviour of talking in front of the patient as if s/he was not present by other staff.

In the surgical area, an increase of 5% in overall satisfaction necessitates addressing all the previously mentioned aspects, along with the behaviour of talking in front of the patient as if s/he was not present by nurses, respect and dignity demonstrated by other hospital staff, and patient involvement. To enhance overall satisfaction by 5% in the medical area, professionals should address the following aspects in addition to those listed earlier: talking in front of the patient as if s/he was not present by doctors, respect and dignity demonstrated by nurses, and patient involvement.

## Discussion

Patient-reported measures are increasingly considered for providing quality health services and for decision making. However, to the best of our knowledge, no previous studies have addressed the question of which experiential aspects of hospitalization should be primarily improved to enhance patient satisfaction, and whether these aspects vary across different hospital settings.

All aspects that matter to patients should be considered for their power of change the patient satisfaction, patient experience and the quality of healthcare services. However, in resource-constrained settings, prioritizing interventions becomes essential. Utilizing patient feedback for quality improvement can be resource-intensive, which is often a barrier to the use of patient-reported data, as noted by several authors [20, 35]. The optimization model employed in this study demonstrates for the first time that patient-reported data can provide detailed insights for managing various aspects of patient experience to enhance overall satisfaction. Applying this kind of methodologies can contribute in identify the first aspects of patient experience to address in a gradual and modular strategy of healthcare services’ improvement, by supporting managers and professionals in identify the priorities in an overwhelming situation of data and knowledge management. Thus, despite we adopted the patient experience as an intermediate result for generating an increased overall satisfaction, the actionability of results regards patient experience, meant also as an outcome per se [3]. Additionally, it highlights the need to disseminate, analyse, and interpret data at different organizational levels to tailor quality improvement actions according to the specific context.

In this study, we considered the experience reported by patients and their overall satisfaction with hospitalization in 7 public facilities in an Italian region (i.e., Tuscany Region). We used data reported by patients discharged from surgical and medical department in 2022, for a total of 24,162 observations.

The departments differ in terms of age and probability of suffering from chronic disease, with individuals discharged from medical wards being older and more likely to be chronic patients, as previously reported in prior studies [22].

We also provide additional findings on the association between patients’ perception of the quality of care and the distinct departmental areas (i.e., surgical and medical) [27]. In particular, our study provides insights into diverse perspectives on the experience with hospital stays in these distinct departments. While the overall satisfaction with the hospitalization experience is similarly reported by patients discharged by surgical and medical departments, the aspect of experience to be prioritized for improving the satisfaction in the two groups are different. This confirms the power of the methodological approach we employed since it aided in pinpointing the optimal blend of experiential factors that result in the intended outcome of satisfaction improvement, by involving maximizing parameters in different settings.

A kind reception upon admission to the department and the pain management are the only aspects that consistently influence overall satisfaction in both surgical and medical areas. Based on the data, the kindness during hospital reception has a significant influence compared to the discharge phase. However, this shouldn’t be assumed as obvious, as the admission phase might involve long waiting time between the different phases from the arrival to the admission procedures and the room allocation, which can be stressful for patients [39]. A kind and efficient reception can alleviate stress, making the overall satisfaction more positive.

Regarding the crucial influence of the pain management on patient satisfaction with hospital stay, our results confirm previous evidence. Other studies have underlined the need of better and more effective pain management for preventing dissatisfaction [36, 37]. This includes the implementation of continuous education on management of pain, particularly in the use of pharmaceutical agents [40].

Additionally, we found that nursing care plays a key role in determining the patient satisfaction in the surgical department. According to Murante et al. [22], surgical patients may have greater pain or other challenges with recovery, and this may require more nursing care during hospitalization [22].

The literature reports that the nursing is among the main determinants of patient satisfaction [41, 42]. This is confirmed by the outcomes of the optimization model for the surgical area, which reports as an early priority working on the patient experience with “Fear and anxiety management by nurses”. Additionally, “Talking in front of the patient as if s/he was not present by nurses” is a determinant of satisfaction to be optimized in the surgical area for reaching the higher percentage of satisfaction improvement, but it is not present among the priority items in the medical area.

In the medical ward, it appears that additional professional not clinical roles also hold significance, as prioritizing their display of respect and dignity towards patients can potentially yield a positive impact on enhancing the overall satisfaction of a medical hospital stay.

Patients in the medical area, generally older and more likely to suffer from chronic conditions, as above-mentioned, may require increased assistance from additional professionals or healthcare assistants in basic patient care, daily activities and personal hygiene. Thus, the interactions that they have with patients can shape the overall experience, as recently found [23].

Some scholars reported that interdisciplinary teamwork positively affected patients’ perceptions of quality of patient-professional communication, person-centred response and continuity of care [43]. Other scholars found that “synergistic teamwork” in the nursing care models is negatively associated with the patient satisfaction [25]. Our findings confirm that the perception of effective teamwork between medical and nursing staff is not among the experience items to be prioritized to increase the overall patient satisfaction.

Other researchers emphasized that physical comfort is among the most important determinants of patient satisfaction [44]. While comfort aspects (i.e., silence, cleanness) are associated with the patient satisfaction, they do not compare among the experience items to be prioritized to improve the overall satisfaction.

In this research, we also found that the experience of a good communication between healthcare professionals and caregivers is statistically significant determinant of satisfaction in the surgical department. This may be due to the fact that patients undergoing surgery, and their caregivers, need clear and precise communication about preoperative and postoperative care. Despite this, the item is not among the factors that should be prioritized to increase the overall satisfaction of patients, likely because greater emphasis is given to the clinical outcome and pain management. Additionally, since our data come from a survey targeted to patients, further studies could be conducted using data directly collected from the voice of caregivers [45]. In future, we could also investigate whether and how the contribution of caregivers during the questionnaire’s completion influences the perception of experience reported by patients.

Overall, this study’s original contribution lies in the demonstration that the statistical tools employed depend on specific problem analysed, serve different purposes, and have distinct outputs. Regression models are used to analyse the magnitude of the association between a dependent variable and one or more independent variables but yield information less suitable for decision-making and resource allocation. Whereas, the optimization models employed in this research, tested in two distinct hospital settings, combine the magnitude of the association with the current level of each item to pinpointing precise aspects of care experience and to directs efforts toward achieving the desired level of improvement in patient satisfaction. Awareness of the experience-related factors that primarily influence overall satisfaction could facilitate a more efficient interpretation and utilization of patient-reported data. This can help in addressing challenges in the data use [20, 35], enhancing quality improvement efforts and fostering value co-creation in healthcare [46]. Furthermore, understanding the relationship between experience and satisfaction in terms of optimization could drive decisions regarding investments and allocation of limited resources (i.e., time, material, economic and human resources).

### Practical implications

This research can support practitioners in their practice of informing quality improvement actions using patient-reported data. The results of the optimization model serve as decision making tools, by providing quantitative insights. The data reported in this research can be used by healthcare managers and practitioners in daily practice for improving patients’ experience and satisfaction, by prioritizing actions and practices and optimizing limited resources.

Patient-reported data are usually skewed towards positive values. Thus, handling patient-data can lead to the perception that only marginal improvements are possible, so undervaluing the utility of such data. Methodologies, such the one adopted in this paper, can integrate other sources of insights for supporting managers and professionals in pinpointing specific experiential areas for targeting their strategy of service improvement. Furthermore, the study’s findings underscore that not all the significant determinants of satisfaction should be the primary focus of improvement actions. Instead, they should be selected through appropriate tools, such as the optimization model or other models, to guide informed managerial decisions with a clear focus on the different settings.

In this study, we used an optimization model, being aware that other methodologies are available. Future studies can employ, for instance, the Kano-model used in Quality Management, or other similar schemes, with their roots in Hertzberg’s 2-factor model used in the hygiene and motivation literature.

### Limitations

Using self-reported data comes with inherent limitations. Since individuals provide the data themselves, there might be instances of questions being misunderstood or varying levels of attention during the survey. However, these challenges are common in all survey-based studies. To address them, we’ve employed translations of standard and widely used items, which helps alleviate these limitations [36]. Additionally, the non-probabilistic nature of the sample and, thus, the issue of representativeness could be considered limitations of the present study. Nevertheless, as stated by Coulter et al. (2009) [9], the above-mentioned aspects do not necessarily reduce the value of surveys’ data, since they can be relevant for quality improvement actions in the operational management approach adopted in this study [45]. Moreover, a strength of this study is the large dataset employed.

Another limitation that opens to future studies is that we considered the two most important areas of hospitalization per size of patients’ population: surgical and medical. Future research could consider other setting of hospitalization, or also other healthcare services, such as primary care services, since the interest in other setting is increasing [47, 48]. Additionally, further studies could analyse factors that affect overall satisfaction, by segmenting patients in groups by sociodemographic characteristics (e.g., age group, gender, level of education), type of hospitalization (ordinary vs emergency, length of stay), type of hospital (e.g., LHA vs TH, dimension, and characteristics of hospital).

In addition, in this study we considered a year (i.e., 2022), because it is difficult to compare different years characterized by important events such as COVID-19 pandemic, which can affect the perception of the experience with the healthcare services [23]. Future studies could address how patient’s experience and satisfaction of patients changes over time, in a longitudinal way.

In addition, our study has the same limitations reported by Seghieri and colleagues (2009) [30]. First, the lack of a cost structure incorporated into the formulation of the optimization model. This implies that the model operates under the assumption that the cost of enhancing each aspect by 1% is uniform. In upcoming studies, there is a need to either investigate the incorporation of a suitable or simulated cost structures to comprehend how this might influence the selection of care aspects. Second, the model was designed for use with continuous variables, while we used data from Likert scales that are ordinal in nature. One potential remedy to bypass the use of averages is to work with distributions. This alternative approach, however, necessitates altering the optimization model formulation and should be regarded as an alternative for future investigations.

## Conclusions

This study is one of the first pieces of research that demonstrates which patient-reported experience factors can be prioritized to enhance the satisfaction level of patients discharged from various hospitalization settings (e.g., surgical and medical departments) through the use of an optimization model. The results proved that, on the one hand, there is a key difference with the results of regression analyses, and, on the other hand, different experiential aspects need to be prioritized for different groups of patients undergoing hospitalization in different departmental areas. This research provides key information on the way in which healthcare managers and professionals could drive their improvement actions for improving patient satisfaction, and eventually enhancing the patient-centredness of the healthcare services. With this research, we want to emphasize the importance going beyond the collection of the patients’ perspective and using their voice in practice employing different tools of analysis, to answer people’s needs in designing and delivery healthcare services.

## Data Availability

The datasets used and analysed that support the findings of this study are not publicly available to maintain patients’ confidentiality, but aggregated data and statistical procedures are available from the corresponding author on reasonable request.

## Abbreviations

LHA: Local Health Authority
PLHAMA: Patient leaving hospital against medical advice
PREMs: Patient-reported experience Measures
SE: Standard Error
TH: Teaching Hospital
